# IgA vasculitis (Henoch – Schönlein Purpura) as the first manifestation of juvenile Systemic Lupus Erythematosus: Case-control study and systematic review

**DOI:** 10.1186/s12887-019-1829-4

**Published:** 2019-11-26

**Authors:** Chiharu Murata, Ana Luisa Rodríguez-Lozano, Hayde Guadalupe Hernández-Huirache, Miriam Martínez-Pérez, Laura Andrea Rincón-Arenas, Esmeralda Nancy Jiménez-Polvo, Francisco Eduardo Rivas-Larrauri, Cecilia Solís-Galicia

**Affiliations:** 1 Research Methodology Department, Instituto Nacional de Pediatría, Ciudad de México, México; 2 Immunology Service, Instituto Nacional de Pediatría, Insurgentes Sur 3700-C, Insurgentes Cuicuilco, Delegación Coyoacán, CP 04530 Mexico City, Mexico; 3 Rheumatology Service, Hospital Regional de Alta Especialidad del Bajío, León, Guanajuato, Mexico; 4 Information and Scientific Documentation Department, Instituto Nacional de Pediatría, Ciudad de México, México

**Keywords:** IgA vasculitis, Henoch-Schönlein Purpura, Systemic lupus erythematosus, Age distribution, Prognostic factors

## Abstract

**Background:**

We have recognized 15 children with jSLE and the antecedent of IgA vasculitis (HSP). This association is not broadly present in the literature.

**Aim:**

To know the age and gender distribution of children with IgA vasculitis (HSP), compare it to our IgA vasculitis (HSP) + jSLE cases, and identify prognostic factors to develop jSLE within our case series, IgA vasculitis (HSP) vs. IgA vasculitis (HSP) + jSLE.

**Methods:**

A s*ystematic review* was carried out to know the age and gender distribution of children with IgA vasculitis (HSP). The information obtained plus data from 110 children with IgA vasculitis (HSP) from the Instituto Nacional de Pediatría were used to compare groups and identify prognostic factors. We performed a c*ase-control study* in patients < 18 years, consisting of 15 cases retrospectively identified with IgA vasculitis (HSP) + jSLE, and 110 IgA vasculitis (HSP) control subjects.

**Results:**

The information of 12,819 IgA vasculitis (HSP) subjects from the systematic review and 110 IgA vasculitis (HSP) controls was obtained and compared to our 15 IgA vasculitis (HSP) + jSLE cases. The mean age of IgA vasculitis (HSP) was 7.1-years vs. 10.4-years of IgA vasculitis (HSP) + jSLE at the HSP diagnosis. Female to male ratio of IgA vasculitis (HSP) was 1:1.33 vs. 1:0.25 of IgA vasculitis (HSP) + jSLE. Patients with IgA vasculitis (HSP) + jSLE had lower levels of Hemoglobin (Hb) compared to patients with IgA vasculitis (HSP) 109 g/L vs. 141 g/L. For the development of jSLE, we found older age and lower levels of Hb as prognostic factors with OR [95% CI]: 1.37 [1.06, 1.89] and 5.39 [2.69, 15.25], respectively.

**Conclusion:**

IgA vasculitis (HSP) + jSLE patients are older and have lower levels of Hb than patients with IgA vasculitis (HSP). It is necessary to confirm these findings through a prospective study.

## Background

IgA vasculitis, formerly Henoch-Schönlein Purpura (HSP) is the most frequent vasculitis in childhood; it is a systemic vasculitis of small vessels [1–3]. The estimated annual incidence is 20.4 per 100,000 children/year [2] though it varies depending on the group of age studied. Aalberse, et al. [3] reported a general incidence of 6.1 per 100,000 children/year, while in the age group between 4 and 7 years-old, the incidence is 70.3 per 100,000 children/year [2]. It is frequently reported in the literature a distinction by group of age, one study including 120 children with IgA vasculitis (HSP) found that children under 10-years represented the 88.3% from the total [4], another study of 107 children, reported that 73% of them were ≤ 10-years [5] and, in a smaller study of 78 patients the percentage of subjects under 10-years reached 90% [6]. In the same articles cited [4–6], we observed a discrete but consistent propensity for male patients, 78 out of 120 (65%), 61 of 107 (57%), and 46 from 78 (59%) were male children.

The classic clinical features of IgA vasculitis (HSP) comprise non-thrombocytopenic purpuric skin rash, abdominal pain, arthralgia or arthritis, and renal involvement, usually with a self-limiting disease course [1]. Skin involvement is the mandatory criterion in patients with IgA vasculitis (HSP) [7]; nevertheless, this is a non-pathognomonic sign of the disease [8]. About 10 % of patients with systemic lupus erythematosus (SLE) can present with cutaneous vasculitis [9]. SLE is a multisystem chronic autoimmune disease [10] antibody-mediated with unpredictable clinical course; usually leading to more severe disease than IgA vasculitis (HSP) [11, 12]. There are few reports in which IgA vasculitis (HSP) and juvenile SLE (jSLE) are somehow associated [13–16].

Herein we describe 15 cases with an initial diagnosis of IgA vasculitis (HSP) who, after a variable period, were diagnosed with jSLE. We hypothesize that these children are demographically different from those children with only IgA vasculitis (HSP) in terms of age and gender.

We performed a systematic review to know the age and gender distribution of children with IgA vasculitis (HSP). To be able to test our hypothesis, that children with IgA vasculitis (HSP) + jSLE are older and with female predominance compared to children with IgA vasculitis (HSP).

Finally, in our patients (*n* = 125, 110 with IgA vasculitis (HSP) and 15 with IgA vasculitis (HSP) + jSLE), we explore the prognostic factors to develop jSLE after IgA vasculitis (HSP).

## Methods

### Systematic review

A Systematic Review of the literature was conducted on PubMed from 1977 to 2016; we include the different terms for naming IgA vasculitis. English and Spanish language, infant, child, and adolescent were selected as limits (Additional file 1). Inclusion criteria for the studies were: a) based on human beings; b) age should be reported as mean and standard deviation, or median with min-max or IQ range; c) gender of all subjects must be specified; d) if the study included children and adults, the children group must meet the formerly mentioned criteria to be included; e) for the case series and literature review, only the index cases were included. Exclusion criteria: a) gender balanced samples; b) those reporting only females or males; c) those referring only complications of IgA vasculitis (HSP); d) articles including subjects with IgA vasculitis (HSP) nephritis exclusively; e) articles published by the same author(s), in which it was suspected or mentioned in methods that these samples are the same, then only the article with the numerous subjects was included, and f) one-case reports. Data extraction was conducted independently by two authors (RLAL and CM). We suspect that patients with IgA vasculitis (HSP) and those with IgA vasculitis (HSP) + jSLE belong to a different population. The information obtained from the systematic review was used exclusively to compare the mean of age distribution and gender proportion with the 15 cases with IgA vasculitis (HSP) + jSLE.

### Case-control study

We carried out a case-control study to test the demographic differences between subjects with IgA vasculitis (HSP) and IgA vasculitis (HSP) + jSLE; we looked for associations between age, gender, and Hb, also we compared the odds ratio (OR) of these variables between groups. Study participants: we retrospectively identified 18 patients with antecedent of IgA vasculitis (HSP) in the INP lupus cohort and three more subjects from the Hospital de Alta Especialidad del Bajío. Case inclusion criteria: a) age < 18 years-old at the IgA vasculitis (HSP) diagnosis; b) initial clinical diagnosis of IgA vasculitis (HSP) according to the EULAR/PRINTO/PReS criteria [17]; c) follow-up from the diagnosis of IgA vasculitis (HSP) to diagnosis of SLE at INP or Hospital del Bajío; d) subsequent diagnosis of SLE according to the 1997 American College of Rheumatology classification criteria [18], patients must have at least four out of 11 classification criteria. Inclusion criteria for the control group: a) clinical diagnosis of IgA vasculitis (HSP) according to the EULAR/PRINTO/PReS criteria or American College of Rheumatology criteria or biopsy; and b) < 18 years-old at IgA vasculitis (HSP) diagnosis. We recruited 110 subjects with IgA vasculitis (HSP) from the INP outpatient clinic to accomplish two functions: one, to contribute to the systematic review, in order to estimate the distribution of age and gender; and two, to compare the distribution of age and gender between IgA vasculitis (HSP) and IgA vasculitis (HSP) + jSLE patients.

### Statistical analysis

Articles from the systematic review reported the age in two different ways, some of them describe mean and standard deviation while others state the median, and the minimum-maximum values, or interquartile range; in these cases, we estimated the mean and the standard deviation (SD) using Hozo’s method [19] for two reasons. One, to obtain all the possible information from articles; and two, to be able to compare age distribution between children with IgA vasculitis (HSP) and children with IgA vasculitis (HSP) + jSLE. For the univariate analysis of numerical variables, mean and SD are described, and proportions for categorical variables. Mean and variance, as well as the gender proportion of each study, were weighted by the sample size. Ninety-five percent confidence interval (95% CI) and Wilcoxon signed-rank test were used to compare the means from the patients with IgA vasculitis (HSP) and IgA vasculitis (HSP) + jSLE. The gender proportion was compared by the 95% CI and *χ*^2^ tests. Finally, the association of the demographic variables with the prognostic factors to develop jSLE after IgA vasculitis (HSP) was analyzed by conditional logistic regression model; the OR and their 95% CI were estimated. All data analysis was done with JMP11, SAS Institute Inc.

## Results

### Systematic review

**Fig. 1:**
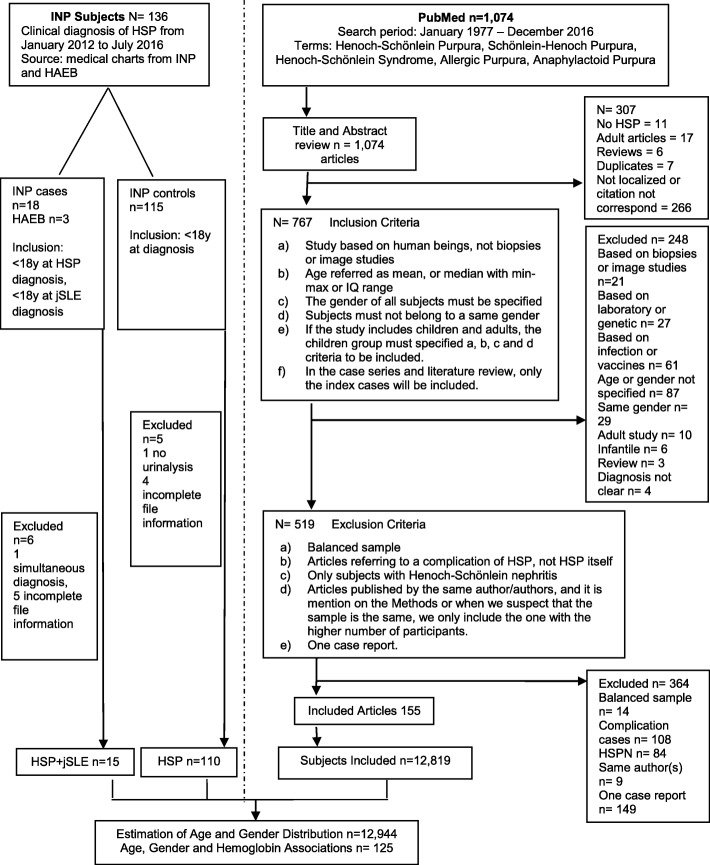
Enrollment, Retrieving, and Analysis Design of the Study

Found articles spanned from the first one published in PubMed in 1977 to December 2016; we include the different terms for naming IgA vasculitis and select English and Spanish language, infant, child, and adolescent as limits (See Additional file 1). A total of 1074 articles were obtained, following title and abstract review 767 articles were selected. After applying the inclusion and exclusion criteria, 155 articles remained for analysis, and provide the information of 12, 819 children (Fig. 1 illustrate the Enrollment, Retrieving, and Analysis Design of the Study. Additional file 2 shows the articles included in the systematic review and Additional file 3 demonstrates the PRISMA Checklist).

The mean age was 7.1-years (SD 2.8) minimum 9 mo – maximum 14.6-years. The age group distribution (< 5, 5–10, >10y) referred to in the article or attained by the data shown by the authors was available for 1615 subjects (12.5% of the total of the sample) as follows: 389, 888 and 338, respectively. Regarding the gender, the male frequency was 7378 (57%), to a female: male ratio of 1:1.33.

### IgA vasculitis (HSP) + jSLE cases

Fifteen subjects from the INP lupus cohort fulfilled the inclusion criteria; the mean age was 10.4-years (SD 3.31), minimum 3y 4 mo – maximum 14.3-years. The age group distribution (< 5, 5–10, >10y) was 1, 7, and 7, respectively, at the IgA vasculitis (HSP) diagnosis. There were three males in the sample for an F: M ratio of 1:0.25. The main clinical features from the 15 cases with IgA vasculitis (HSP) + jSLE are displayed in Table 1.

**Table 1:** Main clinical and laboratory features of patients with IgA vasculitis (HSP) who developed juvenile Systemic Lupus Erythematosus (jSLE)

Pat	G	Age at HSP	Type of skin rash	GI	Joints^a^ (number)	Renal	HSP Recur	Age at SLE	SLE Criteria (ACR)	Renal (g/d)	ANA/ DNA	C3 C4	IgG
1	M	9y 7 m	Purpura	Angina	Knees Ankle (4)	Hem, Prot, Nephrotic	No	9y 10 m	CC, Nephritis, Serositis, Hemolytic A, Lymphopenia	Hem Prot (0.6)	+/+	43.1 2.4	2020
2	F	14y 4 m	Purpura + Petechiae	–	Wrist Ankle (4)	–	yes (1)^b^	15y 2 m	MR, Photosensitivity, Oral ulcers, Arthritis, Nephritis, Lymphopenia	Hem Prot (1.8) Casts	+/+	47.0 4.0	1670
3	F	3y 3 m	Purpura	–	Knees Ankle (2)	Prot Nephrotic	No	3y 6 m	Arthritis, Nephritis, Serositis, Hemolytic A	Prot (0.6)	+/+	17.9 1.6	3240
4	F	12y 4 m	Purpura + Ecchymos	Angina	Knees Ankle (4)	Hem, Prot, Nephrotic	No	12y 6 m	Arthritis, Nephritis	Hem Prot (0.4) Casts	+/+	42.0 6.1	2990
5	F	9y 11 m	Purpura	–	Ankle (1)	Hem	No	10y 1 m	MR, Photosensitivity, Oral ulcers, Arthritis, Nephritis, Hemolytic A, Lymphopenia	Prot (1.3)	+/+	49.9 6.2	620
6	F	13y 7 m	Purpura	Angina Surgery	Knees Ankle Elbow (6)	Hem, Prot, Nephrotic	yes (1)^b^	14y	MR, Oral ulcers, Arthritis, Nephritis, Serositis, Hemolytic A, Lymphopenia	Hem Prot (1.0)	+/+	43.0 7.2	1470
7	F	12y 5 m	Purpura	–	Knees Ankle (2)	Prot Nephrotic	No	12y 7 m	Arthritis, Nephritis, Hemolytic A, Lymphopenia	Prot (0.9)	+/+	24.6 1.5	2580
8	F	13y 2 m	Purpura + Petechiae	–	Knees Ankle Elbow (3)	–	No	13y 5 m	MR, Nephritis	Prot (0.6)	+/+	32.0 1.7	2974
9	F	9y 11 m	Purpura + Ecchymos	Angina	Ankle (1)	Hem, Prot Nephritic	yes (1)	11y 7 m	MR, Photosensitivity, Arthritis, Nephritis	Hem Casts	+/+	55.9 5.6	1170
10	M	11y 11 m	Purpura	–	Ankle (1)	Hem, Prot, Nephrotic	No	12y 2 m	Arthritis, Nephritis, Lymphopenia	Prot (1.5) Casts	+/+	58.5 1.4	2260
11	M	9y 9 m	Purpura	Angina	Knees Ankle Elbow (3)	–	yes (4)^c^	10y 1 m	Arthritis, Nephritis, Hemolytic A, Lymphopenia	Hem Prot (2.0) Casts	+/+	18.3 6.8	2570
12	F	10y 7 m	Purpura	Angina	Ankle (1)	Hem	yes (1)	12y	MR, Oral ulcers, Nephritis, Lymphopenia	Prot (0.6) Casts	+/+	61.2 9.1	1920
13	F	6y 3 m	Purpura	Angina	Knees Ankle (4)	Hem	yes (1)	6y 7 m	Arthritis, Nephritis, Lymphopenia	Hem Prot	+/+	40.0 8.0	1680
14	F	5y 2 m	Purpura	–	Knees Ankle (4)	–	No	7y	MR, Oral ulcers, Arthritis, Nephritis, Hemolytic A, Lymphopenia	Hem Prot Casts	+/+	34.0 3.2	1871
15	F	14y	Purpura	–	Ankle (2)	Nephrotic/ Nephritic	yes (1)	14y 3 m	Arthritis, Nephritis, Lymphopenia	Hem Prot (1.9)	+/+	24.7 1.54	2460

*Pat* Patient identification number, *G* Gender, *Ecchymos* Ecchymosis, *GI* Gastrointestinal involvement, *HSP Recur* HSP Recurrence, *ACR* American College of Rheumatology. ^a^Joints with arthritis or arthralgia; ^b^Persistent purpura; ^c^Cortico-dependence; *Hem* Hematuria, *Prot* Proteinuria (g/d), *Hemolytic A* hemolytic anemia, *MR* Malar Rash, *ANA* Antinuclear antibodies, *DNA* Double stranded deoxyribonucleic acid. C3, C4 and IgG, Reference Values (RV) from local laboratory. C3 RV 111-161 mg/dL. C4 RV 14-42 mg/dL. IgG RV 695-1602 mg/dL

### Group comparison

The comparison of age and gender between subjects with IgA vasculitis (HSP) + jSLE and subjects with IgA vasculitis (HSP), from the systematic review and the INP, showed similar results; that children with IgA vasculitis (HSP) were younger and had a higher proportion of males compared to the 15 cases with IgA vasculitis (HSP) + jSLE (See Table 2).

**Table 2:** Age, gender and hemoglobin comparison in subjects with IgA vasculitis (HSP) versus subjects with IgA vasculitis (HSP) + juvenile Systemic Lupus Erythematosus (jSLE)

	HSP^a^ (*n* = 12,829)	HSP^b^ (*n* = 110)	HSP + jSLE (*n* = 15)	*P*-value HSP^a^ vs. HSP + jSLE	*P*-value HSP^b^ vs. HSP + jSLE
Age [years], mean (SD)	7.1 (2.8)	7.1 (3.5)	10.4 (3.3)	< 0.001^§^	0.002^§^
Gender [F], n (%)	5551 (43%)	61 (55%)	12 (80%)	0.003^¶^	0.060^¶^
Hb [g/L], mean (SD)	–	141 (12)	109 (17)	–	< 0.001^§^

HSP^a^: from the Systematic Review; HSP^b^: from INP; ^§^Wilcoxon’s signed rank test ^¶^ χ^2^ test. This table shows age and gender comparison in subjects with HSP from the systematic review versus subjects with HSP + jSLE from INP; plus the comparison of age, gender and hemoglobin in subjects with HSP versus subjects with HSP + jSLE both from INP

### Case – control study

The effect of age, gender, and hemoglobin (Hb) at the diagnosis time of IgA vasculitis (HSP) on the development of jSLE was analyzed by no-adjusted regression analysis (Table 2), and adjusted regression analysis (Table 3). We observed a significant effect of Hb, which increased, even more, when we dichotomize the variable into Anemia (Hb < 120 g/L) vs. No Anemia (Hb > 120 g/L). Anemia appears to be a prognostic factor for the development of jSLE after IgA vasculitis (HSP) (OR = 66 [95% CI: 16–350]).

**Table 3:** Main characteristics associated with the development of juvenile Systemic Lupus Erythematosus (jSLE) after IgA vasculitis (HSP)

	β (SE)	OR [CI95%]	*P*-value^†^
Gender [F], n (%)	0.07 (0.62)	1.16 [0.08–14.42]	0.907
Age [years], mean (SD)	0.32 (0.14)	1.37 [1.06–1.89]	0.028
Hb [g/L], mean (SD)	1.68 (0.43)	6.07 [3.03–16.95]^a^	< 0.001

*SE* Standard error; ^†^Wald’s Test was performed to assess the whole model significance of the estimated regression coefficient of three independent variables (*P* < 0.001, *R*^2^ = 0.73). ^a^OR was calculated per unit (10 g/L) of Hb decreased

## Discussion

This study is the first to characterize the age and gender distribution of patients with IgA vasculitis (HSP) based on systematic review and meta-analysis. These results allowed us to confirm that patients with IgA vasculitis (HSP) developed the disease at an early age, with a mean age of about 7 years. A predominance of boys affected by IgA vasculitis (HSP) was also observed in the meta-analysis, contrasting with the 15 patients reported herein with IgA vasculitis (HSP) + jSLE, who were older and had a female majority. The association analysis in our sample of 125 subjects permitted us to identify age and Hb as prognostic factors associated with the subsequent development of jSLE.

Anemia or hemoglobin levels are not reported systematically in the studies of patients with IgA vasculitis (HSP), also in different studies, the value to determine anemia is not consistent. Calvo-Río et al. [7], compared their 417 patients and a series of children and adults with IgA vasculitis (HSP), they found 8.9% of anemia (Hb < 110 g/L) in their cases, compared to three of six studies that also found anemia, 2.3% in 87 patients, 7.7% in 104 and 1.6% in 61 patients. Trapani et al. [20], reported a higher frequency of anemia, 14% of 150 patients. If patients with IgA vasculitis (HSP) nephritis were included, the frequency of anemia increased up to 25.5% [21]. Surprisingly, in our sample of patients with sole IgA vasculitis (HSP), we did not identify any patient with hemoglobin level ≤ 110 g/L, while it was present in 53% of patients with IgA vasculitis (HSP) + jSLE. We did not foresee the role of hemoglobin as a prognostic factor in patients with IgA vasculitis (HSP); it could represent one of the first clinical indicators of a progression of the disease to IgA vasculitis (HSP) + jSLE. Although, there might be patients with IgA vasculitis (HSP) in our sample that already had jSLE, the initial clinical presentation in all of them was IgA vasculitis (HSP). The idea we would like to highlight is that some patients with IgA vasculitis (HSP) could develop jSLE and that IgA vasculitis (HSP) could be the first manifestation of jSLE.

There are only four case-reports published in the medical literature in which the association between IgA vasculitis (HSP) and jSLE is present [13–16]. The case described by Al-Attrach [14] is particularly interesting due to the similarity to our cases. The patient was a 12-year-old girl diagnosed with IgA vasculitis (HSP) based on skin, joint, abdominal, and renal affection, after 5 months on treatment with corticosteroids and mycophenolate mofetil, she attained withdrawal of medications. One month later, she developed pleural and pericardial effusion, increase of proteinuria and positive ANA and ds-DNA antibodies; she was then diagnosed with jSLE. There was also a 9-year-old girl diagnosed with lupus, who after 3 months, returned to the hospital with abdominal pain and purpuric rash [16]. The other two cases [13, 15] were two 13-years-old boys with purpura and nephritis. One of them also developed serositis; the autoantibodies were negative in both cases; as a result, one case was reported as IgA vasculitis (HSP) with lupus-like nephritis, and the other one as ANA-negative SLE and full-house nephropathy.

The origin of this research was a 7-year-old girl attended in 2006, she was diagnosed with appendicitis and underwent surgery, which showed a swollen small intestine with leukocytoclastic vasculitis on the biopsy. She continued with abdominal pain, and a few days later a skin rash appeared over her lower extremities, it was palpable purpura; she developed proteinuria and hematuria and was diagnosed with IgA vasculitis (HSP). Despite steroid treatment for several weeks, she presented seizures and the worsening of proteinuria. The suspicion of SLE rose, the renal biopsy was compatible with lupus nephritis class II-b (WHO classification), ANA and ds-DNA autoantibodies were positive, thus confirming jSLE diagnosis (case not included in this case series).

On the topic of autoantibodies in IgA vasculitis (HSP), some articles discuss the importance of the positivity of anti-nuclear antibodies, one of them refers to the persistence of the ANA for as long as 6 years regardless of disease activity [22]. The other study questions the relevance of ANA in the setting of IgA vasculitis (HSP), arguing that up to 18% of healthy children may have positive ANA [23].

IgA vasculitis (HSP) has been referred to as a benign disease [4, 21]; the long term outcome is related to the presence of renal involvement [7, 24, 25]. The initial clinical manifestations of IgA vasculitis (HSP) and jSLE as arthritis, mild to severe renal affection, and skin rashes could be seen in both diseases. However, it is not expected that a patient diagnosed with IgA vasculitis (HSP) developed clinic and laboratory features of jSLE. To the best of our knowledge, this is the first case-series reporting IgA vasculitis (HSP) as the first manifestation of jSLE.

At least theoretically, we can consider two different scenarios to explain the connection between IgA vasculitis (HSP) and jSLE. The first one, in which there would be no etiopathogenic link, thus comprising two distinct populations: a) patients with IgA vasculitis (HSP) characterized by a predominance of males and with mean age about 7-years; and b) patients with IgA vasculitis (HSP) onset around 10-years with a higher proportion of females who develop jSLE afterward. This scenario fits quite well with our results; male younger patients had IgA vasculitis (HSP), as we observed in the systematic review, and older females having IgA vasculitis (HSP) as the first manifestation of jSLE, as observed in our cases.

However, it seems to be a connection between these diseases, leading us to the second scenario, in which some patients with IgA vasculitis (HSP) would develop jSLE; it is a possibility that some patients with IgA vasculitis (HSP) also have an underlying defect in complement system and/or defective clearance of apoptotic cells, thus having an infection or being exposed to other factors would allow the formation of immune complexes that will lead to the autoreactive B cells to turn into autoantibodies-secreting plasma cells, giving place to a persistent state of autoimmunity. Nevertheless, we found difficult to explain our results with this second scenario. The question remaining is how to elucidate the demographic differences (age and gender) with the development of jSLE?

Our study has several limitations. The retrospective design implies the risk of bias, being our principal concern inclusion bias. If that is the case, either the patient with IgA vasculitis (HSP) has jSLE since the beginning, or IgA vasculitis (HSP) is, in fact, the initial manifestation of jSLE.

Regarding the limitations from the systematic review, we did not review 266 articles, as some citations did not correspond to the articles we were looking for, or they were published before 1980 and could not obtain the full version. Cases reported in the systematic review were heterogeneous; therefore, we cannot discard that some of those patients have developed lupus. Also, we did not have a methodologically, pure control group. In this situation, as patients with IgA vasculitis (HSP) + jSLE are older and predominantly girls, the direction of the bias should reduce the contrast between groups. However, the differences observed regarding the age and gender distribution are noticeable. We believe that these findings should not be ignored and, that our hypothesis (IgA vasculitis (HSP) and IgA vasculitis (HSP) + jSLE patients are from different populations) is worth to be tested through a prospective study, as it would allow us to identify patients with associated factors to develop jSLE.

We are aware that this is a small study and despite our limitations, found the empirical basis that supports our observation, that children with IgA vasculitis (HSP) + jSLE are older, and have a female predominance. We also observe a statistical difference in the age and hemoglobin levels among cases (15 patients with IgA vasculitis (HSP) + jSLE) and controls (110 patients with IgA vasculitis (HSP). We consider these our meaningful strengths.

Our discussion based on the systematic review enables us to build the theoretical scenarios described above. For these reasons, we claim that patients, especially girls with IgA vasculitis (HSP) who debut at an older age, and with lower levels of hemoglobin, require closer follow-up of the disease.

This is the first step to elucidate the possible relationship between IgA vasculitis (HSP) and jSLE. A prospective and protocolize study of IgA vasculitis (HSP) cases will yield important information and will contribute to the better care of these patients.

## Conclusion

Patients who developed jSLE after IgA vasculitis (HSP) are older, with lower levels of Hb and predominantly females. It is necessary to confirm these findings through a prospective study.

## Supplementary information

**Additional file 1.** Search Terms, this file shows the search strategy carried out in Pub Med.

**Additional file 2.** Systematic Review Articles, it shows the description of each article included in the systematic review, as the Authors, year of publication, title, number and gender of the patients included.

**Additional file 3.** PRISMA checklist.

## Data Availability

The dataset used and analysed during the current case-control study is available from the corresponding author on reasonable request. All data generated and analysed for the systematic review of this study are included in this published article as supplementary information, see Additional file 2.

## Abbreviations

ANA: Anti-nuclear antibodies
ds-DNA: Double stranded deoxyribonucleic acid
Hb: Hemoglobin
HSP: Henoch-Schönlein purpura
jSLE: Juvenile systemic lupus erythematosus
