# Supplementary material for: IgA vasculitis (Henoch – Schönlein Purpura) as the first manifestation of juvenile Systemic Lupus Erythematosus: Case-control study and systematic review
Source: BMC Pediatr. 2019 Nov 26;19:461. doi: 10.1186/s12887-019-1829-4 (PMC6878691; doi:10.1186/s12887-019-1829-4)
Supplement: Supplementary file 1 — Additional file 1. Search Terms, this file shows the search strategy carried out in Pub Med. [file 12887_2019_1829_MOESM1_ESM.docx]

**Additional File 1. Search terms used for the systematic review**

The Systematic Review of the literature was conducted on PubMed from 1977 to 2016:

(("purpura, schoenlein-henoch"[MeSH Terms] OR ("purpura"[All Fields] AND "schoenlein-henoch"[All Fields]) OR

"schoenlein-henoch purpura"[All Fields] OR ("henoch"[All Fields] AND

"schonlein"[All Fields] AND

"purpura"[All Fields]) OR "henoch schonlein purpura"[All Fields]) AND

((Journal Article[ptyp] OR Case Reports[ptyp]) AND

hasabstract[text] AND

("1977/01/01"[PDAT] : "2016/12/31"[PDAT]) AND

"humans"[MeSH Terms] AND

(English[lang] OR Spanish[lang]) AND

("infant"[MeSH Terms] OR "child"[MeSH Terms] OR "adolescent"[MeSH Terms]))
