# Supplementary material for: IgA vasculitis (Henoch – Schönlein Purpura) as the first manifestation of juvenile Systemic Lupus Erythematosus: Case-control study and systematic review
Source: BMC Pediatr. 2019 Nov 26;19:461. doi: 10.1186/s12887-019-1829-4 (PMC6878691; doi:10.1186/s12887-019-1829-4)
Supplement: Supplementary file 2 — Additional file 2. Systematic Review Articles, it shows the description of each article included in the systematic review, as the Authors, year of publication, title, number and gender of the patients included. [file 12887_2019_1829_MOESM2_ESM.docx]

| **Additional File 2. Systematic Review Table of Included Studies** | | | | |
| --- | --- | --- | --- | --- |
| **Author, year** | **Title** | **Pat #** | **Age** | **M - F** |
| Trapani S, 2010 (1) | Severe hemorrhagic bullous lesions in Henoch Schönlein purpura: Three pediatric cases and review of the literature | 3 | 9 y, 11 y, 7 y | F, M, F |
| Kawasaki K, 2006 (2) | Factor XIII in Henoch-Schönlein purpura with isolated gastrointestinal symptoms | 3 | 10, 7, 8 | M, M, F |
| Gultekin A, 1989 (3) | Intestinal Obstruction Due to Henoch-Schönlein Purpura | 3 | 8, 9, 9 | F, M, M |
| Belman A, 1985 (4) | Neurologic manifestations of Schoenlein-Henoch purpura: report of three cases and review of the literature | 3 | 7y6mo, 7, 13 | M, F, M |
| Somekh E, 1983 (5) | Muscle involvement in Schönlein-Henoch syndrome | 3 | 11, 5y6m, 9 | M, F, M |
| Otieno L, 1982 (6) | Schönlein-Henoch Syndrome with Nephropathy | 3 | 14, 8, 7 | 2 – 1 |
| Zhang Y, 2008 (7) | Sibling cases of Henoch-Schönlein purpura in two families and review of literature | 4 | 15, 7, 16, 18y | M M F M |
| Chen Y, 2012 (8) | Familial cases of Henoch-Schönlein purpura in Taiwanese aborigines | 6 | 6 y, 6 y, 8 y, 14 y, 11 y, 8 y, | F, M, M, M, F, M, |
| Casonato A, 1996 (9) | Abnormally large von Willebrand factor multimers in Henoch-Schönlein purpura | 6 | 18,19,16,14,15, 6 (14.6mean) | M, M, M, M, M, F |
| Sönmez K, 2002 (10) | Conservative treatment for small intestinal intussusception associated with Henoch-Schönlein's purpura | 7 | 5.8 (3 – 9) | 5 – 2 |
| Kato S, 1992 (11) | Gastrointestinal endoscopy in Henoch-Schönlein purpura | 7 | 13, 8, 4, 9, 7, 5, 5 | 2M, F,4M |
| Coovadia HM, 1982 (12) | Henoch-Schönlein purpura in Black and Indian children in Natal | 8 | 7.1 (5 -12) | 5 – 3 |
| Shin J, 2005 (13) | Role of mesangial fibrinogen deposition in the pathogenesis of crescentic Henoch-Schönlein nephritis in children | 9  12* | 7.4 ± 1.3  10.2 ± 0.9 | 6 – 3  9 – 3 |
| Tomomasa T, 1987 (14) | Endoscopic Findings in Pediatric Patients with Henoch-Schönlein Purpura and Gastrointestinal Symptoms | 9 | 9.6 (4-14) | 7 – 2 |
| Shin J, 2011 (15) | The gene expression profile of matrix metalloproteinases and their inhibitors in children with Henoch-Schönlein purpura | 10 | 6.02 y (2.9 – 11.7) | 6 – 4 |
| Motoyama O, 2005 (16) | Henoch-Schönlein purpura with hypocomplementemia in children | 10 | 6.2 (2-10) | 2 – 8 |
| Dursun I, 2011 (17) | Circulating endothelial microparticles in children with Henoch-Schönlein purpura; Preliminary results | 11  9* | 8.7 ± 3.1  10.1 ± 3.9* | 6 – 5  4 – 5 |
| Harada T, 2011 (18) | Superior mesenteric artery syndrome: Risk factor for duodenal involvement in Henoch-Schönlein purpura | 12 | 6 y (3 – 13) | 8 – 4 |
| Kawasaki Y, 2005 (19) | Possible pathologenic role of interleukin-5 and eosino cationic protein in Henoch-Schönlein purpura nephritis | 12  20* | 7.7 ± 1.6  8.1 ± 2.7 | 7 – 5  12 – 8 |
| Mahajan V, 2009 (20) | Serum and urine nitric oxide levels in children with Henoch-Schonlein purpura during activity and remission: A study from north India | 14 | 5.4 ± 2.98 y  (0.8 – 11) | 9 – 5 |
| Salhan M, 2007 (21) | Antiphospholipid antibodies in children with Henoch–Schönlein purpura: a prospective study from North India | 14 | 6.3 y (1.3 – 11) | 13 – 1 |
| Nishida M, 1999 (22) | Serum hepatocyte growth factor levels in Henoch-Schönlein purpura | 14acute  17recover | 7.8 (2-13)  7.5 (2-22) | 8 – 6  8 – 9 |
| Couture A, 1992 (23) | Evaluation of abdominal pain in Henoch-Schönlein syndrome by high frequency ultrasound | 14 | 6.85 (20mo-12y) | 9 – 5 |
| Tönshoff B, 1992 (24) | Increased biosynthesis of vasoactive prostanoids in Schönlein-Henoch purpura | 14 | 6 med (2.5-16) | 10 – 4 |
| Kagimoto S, 1993 (25) | Duodenal Findings on Ultrasound in children with Schonlein-Henoch Purpura and Gastrointestinal Symptoms | 14 | 6.92 (4-12) | 8 – 6 |
| Soylu A, 2010 (26) | TLR-2 Arg753Gln, TLR-4 Asp299Gly, and TLR-4 Thr399Ile polymorphisms in Henoch Schönlein purpura with and without renal involvement | 15  15* | 7.1 ± 3.3  8.1 ± 3.5 | 8 – 7  9 – 6 |
| Motoyama O, 2005 (27) | Familial cases of Henoch-Schönlein purpura in eight families | 15 | 6.13 ( 3-10) | 8 – 7 |
| Cazzato S, 1999 (28) | Pulmonary function abnormalities in children with Henoch-Schönlein purpura | 15 | 7.6±1.9 (5-13) | 8 – 7 |
| Gattorno M, 1998 (29) | Differences in Tumor Necrosis Factor-alpha Serum Concentrations Between Patients with HSP and Pediatric Systemic Lupus Erythematosus: Pathogenic Implications | 16 | 5.7 (2.5-12) | 10 – 6 |
| Demircin G, 1998 (30) | Erythrocyte superoxide dismutase activity and plasma malondialdehyde levels in children with Henoch Schönlein purpura | 16 | 8.5 ± 0.68  (3-13) | 7 – 9 |
| De Mattia D,1995 (31) | von Willebrand factor and factor XIII in children with Henoch-Schönlein purpura | 16 | 7 mean (3-11) | 7-9 |
| Farley T, 1989 (32) | Epidemiology of a Cluster of Henoch-Schönlein Purpura | 16 | 5 med (2-9) | 10 – 6 |
| Brendel-Müller K, 2001 (33) | Laboratory signs of activated coagulation are common in Henoch-Schönlein purpura | 17 | 5.6 mean  (3-13) | 8 – 9 |
| Henriksson P, 1977 (34) | Factor XIII (Fibrin stabilising Factor) in Henoch-Schönlein Purpura | 17 | 6.1 (2.5 – 13) | 12 – 5 |
| Tsuji Y, 2004 (35) | Urinary leukotriene E4 in Henoch-Schönlein purpura | 18 | 6.59 ± 2.57  (4 – 15) | 6 – 12 |
| Saulsbury F, 1983 (36) | Thrombocytosis in Henoch-Schönlein Purpura | 18 | 4.5 (10m-11y) | 11 – 8 |
| Fuentes Y, 2014 (37) | Urinary MCP-1/creatinine in Henoch-Schönlein purpura and its relationship with nephritis | 20  57* | 4, 15, 1  4, 41, 12 | 12 – 8  32 – 25 |
| Mahajan N, 2013 (38) | Levels of interleukin-18 and endothelin-1 in children with Henoch-Schönlein purpura: A study from Northern India | 20 | 5 y (med)  3 – 12y | 12 – 8* |
| Pelliccia P, 1999 (39) | Elevated levels of circulating immunostimulatory 90K in Henoch-Schoenlein purpura | 20 | 5.8 ± 2.8 | 11 – 9 |
| Moja P, 1998 (40) | Is there IgA from gut mucosal origin in the serum of children with Henoch-Schönlein purpura? | 20  12* | 6.5 (3 – 12)  9 (6 – 16) | 13 – 7  5 – 7 |
| Soylemezoglu O, 1996 (41) | Circulating adhesion molecules ICAM-1, E-selectin, and von Willebrand factor in Henoch-Schönlein purpura | 20 | 9.3med(3-15) | 11-9 |
| Özdemir H, 1995 (42) | Sonographic demonstration of intestinal involvement in Henoch-Schönlein syndrome | 20 | 10.5 (5-15) | 13 – 7 |
| Huber A, 2004 (43) | A randomized, placebo-controlled trial of prednisone in early Henoch Schönlein Purpura | 21Pred  19Plac | 5 med(2-11)  6.1med(3-15) | 13 – 8  12 – 7 |
| Cayci FS, 2015 (44) | An Analysis of the Levels of the Soluble Form of the Endothelial Protein C Receptor in Children with Henoch-Schönlein Purpura. | 22 | 8 ± 2.3 (5-13) | 15 - 7 |
| Xie J, 2015 (45) | Expansion of Circulating T Follicular Helper Cells in Children with Acute Henoch-Schönlein Purpura | 22 | 8.27 ± 2.57 | 13 -9 |
| Aliyazicioglu Y, 2007 (46) | Leptin levels in Henoch-Schönlein purpura | 22 | 8.5 med (3 -16) | 14 – 8 |
| Allen A, 1998 (47) | Abnormal IgA glycosylation in Henoch-Schönlein purpura restricted to patients with clinical nephritis | 22  24* | 5.5 (3-13)  7.0 (3-12) | 10 – 12  14 – 10 |
| Glasier C, 1981 (48) | Henoch-Schönlein Syndrome in Children: Gastrointestinal manifestations | 22 | 5y6m (20mo-11 y) | 10 -12 |
| Yilmaz A, 2009 (49) | Effect of paraoxonase 1 gene polymorphisms on clinical course of Henoch-Schönlein purpura. | 24  22* | 7.3 ± 2.6  8.5 ± 2.7 | 15 – 9  10 – 12 |
| Del Vecchio G, 2008 (50) | Cytokine Pattern and Endothelium Damage Markers in Henoch-Schönlein Purpura | 24 | 6 y (med) 3-14 | 9 – 15 |
| Fujieda M, 1998 (51) | Soluble thrombomodulin and antibodies to bovine glomerular endothelial cells in patients with Henoch-Schönlein purpura | 25 | 8.3 (4-15) | 14 – 11 |
| Tahan F, 2007 (52) | The role of chemokines in Henoch Schönlein Purpura | 26 | 7.2 ± 0.4 | 17 – 9 |
| Besbas N, 1998 (53) | Thrombomodulin, tissue plasminogen activator and plasminogen activator inhibitor-l in Henoch-Schönlein purpura | 26 | 9.1 (4-15) | 14 - 12 |
| Ece A, 2008 (54) | Antioxidant enzyme activities, lipid peroxidation, and total antioxidant status in children with Henoch-Schönlein purpura | 29 | 9.3±2.7 y  9med(6 – 15y) | 16 -13 |
| Yang Y, 2004 (55) | The association between transforming growth factor-β gene promoter C-509T polymorphism and Chinese children with Henoch-Schönlein purpura | 29 | 6.5 ± 3.0  (1 – 14.2) | 15 – 14 |
| Chaussain M, 1992 (56) | Impairment of lung diffusion capacity in Schönlein-Henoch purpura | 29 | 7.31 (4 -11.8) | 15 – 14 |
| Li Y, 2012 (57) | Investigation of the change in CD4+ T cell subset in children with Henoch-Schönlein purpura | 30 | 5.93 ± 1.48  (3.02– 10.35y) | 17 – 13 |
| Wang Y, 2011 (58) | Imbalance of interleukin-18 and interleukin-18 binding protein in children with Henoch-Schönlein purpura | 30  19 | 4 y (2 – 9 y)  5 y (4 – 11y) | 19 – 11  11 – 8 |
| Fessatou S, 2008 (59) | Endothelin 1 levels in relation to clinical presentation and outcome of Henoch Schönlein purpura | 30 | 6.3 ± 3 (2 – 12.6) | 14 – 16 |
| Grover N, 2007 (60) | A five year review of clinical profile in HSP | 30 | 10.5 ± 3.95  (3.5 – 17y) | 19 – 11 |
| Muslu A, 2002 (61) | Endothelin levels in Henoch-Schönlein purpura | 30 | 9.7 ± 3.4 (2.5-16) | 17 – 13 |
| Kauffmann R, 1980 (62) | Circulating IgA-Immune Complexes in Henoch-Schönlein Purpura | 30 | 6.1 (0.5-13) | 16 – 14 |
| Koskimies O, 1981 (63) | Henoch-Schönlein Nephritis: Long-Term Prognosis of Unselected Patients | 31 | 8.4 (2.5-14.1) | 17 – 14 |
| Qin W, 2011 (64) | Increased OX40 and soluble OX40 ligands in children with Henoch-Schönlein purpura: Association with renal involvement | 32 | 7.4 ± 1.9 y | 21 – 11 |
| Zampetti A, 2009 (65) | Longitudinal study of microvascular involvement by nailfold capillaroscopy in children with Henoch-Schönlein purpura | 32 | 7.5 ± 3.4  (3 – 16) | 18 – 14 |
| Johnson EF, 2014 (66) | Henoch-Schönlein purpura and systemic disease in children: Retrospective study of clinical findings, histopathology and direct immunofluorescence in 34 paediatric patients | 34 | 10.7 | 14 - 20 |
| Ge W, 2014 (67) | Pentraxin 3 as a novel early biomarker for the prediction of Henoch-Schönlein purpura nephritis in children | 34  37* | 6.78 ± 2.73  6.97 ± 2.17 | 16 – 18  18 – 19 |
| Eisenstein E, 2006 (68) | Analysis of a uteroglobin gene polymorphism in childhood Henoch-Schonlein purpura | 34 | 4.2  5mo – 12 y | 24 – 10 |
| Topaloglu R, 2001 (69) | Vascular Endothelial Growth Factor in Henoch-Schönlein Purpura | 34 | 9.2±0.5 (4-15) | 19 – 15 |
| Chen T, 2011 (70) | Elevated serum heme oxygenase-1 and insulin-like growth factor-1 levels in patients with Henoch-Schönlein purpura | 36 | 11.75± 3.96 y | 17 – 19 |
| Ozaltin F, 2003 (71) | The role of apoptosis in childhood Henoch-Schönlein purpura | 37 | 9±3.3 (2.5 -17) | 22 – 15 |
| Saulsbury F, 1998 (72) | Increased Serum IgD concentrations in children with Henoch-Schönlein purpura | 39 | 6.3 ± 2.6 | 19 – 20 |
| Blanco Quiros, 1994 (73) | Anti-immunoglobulin antibodies in children with Schönlein-Henoch syndrome. Absence of serum anti-lgA antibodies | 39 | 6y8m(3-12) | 19 – 20 |
| Inoue C, 2008 (74) | Efficacy of early dental and ENT therapy in preventing nephropathy in pediatric Henoch-Schönlein purpura | 40 | 6.7 ± 2.5 y (3 – 14y) | 21 – 19 |
| Al Rasheed S, 1991 (75) | Henoch-Schönlein Syndrome in Saudi Arabia | 40 | 6.9 (5m-13y) | 23 – 17 |
| Emre S, 2011 (76) | Methylenetetrahydrofolate reductase C677T polymorphism in patients with Henoch-Schönlein purpura | 41 | 7.8 ± 2.9 y | 25 – 16 |
| Nchimi A, 2008 (77) | Significance of bowel wall abnormalities at ultrasound in Henoch-Schönlein purpura | 43 | 6.3 ± 2.3 y  (3 – 14) | 24 – 19 |
| Yildiz B, 2008 (78) | Increased serum levels of insulin-like growth factor (IGF)-1 and IGF-binding protein-3 in Henoch-Schönlein purpura | 44 | 8.6 ± 3.9 y | 30 – 14 |
| Kumar L, 1998 (79) | Henoch-Schönlein purpura: The Chandigarh experience | 45 | 7.6 (2.5-12) | 30 – 15 |
| Ilan Y, 1991 (80) | Schönlein- Henoch Syndrome in Adults and Children | 46 | 7y (4mo – 14) | 22 – 24 |
| Bagga A, 1991 (81) | Henoch-Schonlein Syndrome in Northern Indian Children | 47 | 8.5±2.8 (3-12) | 34 – 13 |
| Spasojević-Dimitrijeva B, 2011 (82) | Henoch-Schönlein purpura outcome in children: Ten-year clinical study | 49 | 7.62 ± 4.68 y | 30 – 19 |
| Mao Y, 2012 (83) | Urinary angiotensinogen levels in relation to renal involvement of Henoch-Schönlein purpura in children | 51  43 He  13 Pr | 6.72 1 2.36 y  6.65 1 2.34 y  6.58 1 2.42 y | 24 – 27  21 – 22  5 – 8 |
| Dagan E, 2006 (84) | Henoch-Schönlein purpura: Polymorphisms in thrombophilia genes | 52 | 6.7±2.4  6 med (2-14) | 32 – 20 |
| Gershoni-Baruch R, 2003 (85) | Prevalence and significance of mutations in the familial mediterranean fever gene in Henoch-Schönlein Purpura (Retrospective, Michel criteria) | 52 | 6.7 ± 2.4 | 34 – 18 |
| Albaramki J, 2016 (86) | Henoch-Schönlein Purpura in Childhood a Fifteen-Year Experience at a Tertiary Hospital | 55 | 7 ± 3.02 (1-12) | 33 - 22 |
| Alfredo C, 2007 (87) | Henoch-Schönlein purpura: recurrence and chronicity | 55 | 6.0 ± 2.5 | 24 – 31 |
| Salah S, 2014 (88) | MEFV gene mutations in Egyptian children with Henoch-Schönlein purpura. | 60 | 8.1 ± 3 y | 29 - 31 |
| Nickavar A, 2012 (89) | Clinicopathologic correlations in Henoch-Schönlein nephritis | 64  41* | 5.3 ± 2.64  7.2 ± 2.57 | 38 – 26  23 – 18 |
| Smith GC, 1997 (90) | Complement activation in Henoch-Schönlein purpura | 64 | 6.41(1.6-13.87) | 31-33 |
| Hung S, 2009 (91) | Clinical Manifestations and Outcomes of Henoch-Schönlein Purpura: Comparison between Adults and Children | 65 | 6.89 ± 3.69 | 35 – 30 |
| Kanik A, 2015 (92) | Faecal calprotectin levels in children with Henoch–Schönlein purpura: is this a new marker for gastrointestinal involvement? | 66 | 7.5 ± 2.9 y | 29 - 37 |
| Pisacane A, 1992 (93) | Infant Feeding and Henoch-Schönlein Purpura | 67 | 6.08 (2.6) | 36 – 31 |
| Qin Y, 2011 (94) | Cut-off values for serum matrix metalloproteinase-9: Is there a threshold to predict renal involvement for Henoch-Schönlein purpura in children? | 68  66* | 6.63 ± 2.37 y  6.28 ± 2.46 y | 33 – 35  32 – 34 |
| Hamdan J 2008 (95) | Henoch-Schönlein purpura in children. Influence of age on the incidence of nephritis in children | 68 | 5.9 y (mean) (16m-13y) | 40 – 28 |
| Martínez López M, 2007 (96) | Púrpura de Schönlein-Henoch. Estudio de factores asociados con el desarrollo y evolución de la enfermedad | 70 | 6.5 ± 2.7 | 43 – 27 |
| Wang X, 2016 (97) | Henoch-Schönlein purpura with joint involvement: Analysis of 71 cases | 71 | 8.55 ± 2.13 | 40 - 31 |
| García-Porrúa C, 2002 (98) | Henoch-Schönlein purpura in children and adults: Clinical differences in a defined population | 73 | 6.1 ± 3.1 | 34 – 39 |
| Lardhi A, 2012 (99) | Henoch-Schönlein purpura in children from the eastern province of Saudi Arabia | 78 | 6.3 (1.8 – 12) | 46 - 32 |
| Ding W, 2010 (100) | Urinary excretion and renal production of hepatocyte growth factor in children with Henoch-Schönlein purpura | 78 | 8.2 y (6 – 18) | 51 – 27 |
| Calviño M, 2001 (101) | Henoch-Schönlein purpura in children from northwestern Spain: a 20-year epidemiologic and clinical study | 78 | 6.2±3.1 | 36 – 42 |
| Elmas A, 2016 (102) | Platelet Counts in Children with Henoch–Schönlein Purpura—Relationship to Renal Involvement *Nephritis | 79/107  28/107* | 7.1 ± 2.3  8.7 ± 4.1 | 42 – 37  19 – 9 |
| Wu S, 2014 (103) | Add-on therapy with montelukast in the treatment of Henoch-Schönlein purpura | 84  46* | 6.7 ± 1.6  7.25 ± 1.9* | 53 - 31  28 – 18* |
| Ronkainen J, 2006 (104) | Early prednisone therapy in Henoch-Schönlein purpura: a randomized, double-blind, placebo-controlled trial | 84 pred  87 plac | 6.8 (2-15.2)  7.3 (1.7-15.6) | 49 – 35  44 – 43 |
| Lin S, 1988 (105) | Henoch-Schönlein purpura in Chinese children and adults | 84 | 6.6 ± 2.7 | 54 – 30 |
| Yang Y, 2012 (106) | Identification and characterization of IgA antibodies against B2-glycoprotein I in childhood Henoch-Schönlein purpura | 88 | 6.7 y  (2.7 – 17) | 50 - 38 |
| Teng X, 2016 (107) | Evaluation of serum procalcitonin and C-reactive protein levels as biomarkers of Henoch-Schönlein purpura in pediatric patients | 89 | 6.91 (3 – 13) | 54 - 35 |
| Cohen N, 2015 (108) | Predictors of hospital length of stay in pediatric Henoch-Schönlein purpura | 89 | 6.0 ± 0.34 (9mo-17y) | 50 - 39 |
| Rigante D, 2005 (109) | Predictive factors of renal involvement or relapsing disease in children with Henoch-Schönlein purpura | 94 | 6.33 ± 2.54 (3 – 14) | 46 – 48 |
| Kawasaki Y, 2003 (110) | Clinical and pathological features of children with Henoch-Schönlein purpura nephritis: risk factors associated with poor prognosis | 94  20* | 8.7 ± 3.1  10.4 ± 4.7 | 44 – 50  8 – 12 |
| Xu H, 2014 (111) | Interferon-Gamma Gene Polymorphism + 874 ( A / T ) in Chinese Children with Henoch-Schönlein Purpura | 97 | 6.5 ± 2.2  7.6 ± 2.8 | 55 M  42 F |
| Soylemezoglu O, 2008 (112) | CTLA-4 +49 A/G genotype and HLA-DRB1 polymorphisms in Turkish patients with Henoch-Schönlein purpura | 100 | 8.26 ± 3.58 y | 58 – 42 |
| Saulsbury F, 1999 (113) | Henoch-Schönlein purpura in children. Report of 100 patients and review of the literature | 100 | 5.9±2.9 | 57 – 43 |
| Watson L, 2012 (114) | Henoch Schönlein purpura - A 5-year review and proposed pathway | 102 | 6.3 (med) | 49 – 53 |
| Bayram C, 2011 (115) | Prevalence of MEFV gene mutations and their clinical correlations in Turkish children with Henoch-Schönlein purpura | 107 | 7.2 ± 2.5 | 57 – 50 |
| Shin J, 2011 (116) | Intravenous dexamethasone followed by oral prednisolone versus oral prednisolone in the treatment of childhood Henoch–Schönlein purpura | 111 | 6.9 ± 2.3 y | 54 – 57 |
| Kang Y, 2014 (117) | Differences in clinical manifestations and outcomes between adult and child patients with Henoch-Schönlein purpura. | 112 | 7.5 ± 4.4  (1 – 20y) | 64 - 48 |
| Özkaya O, 2006 (118) | Renin-angiotensin system gene polymorphisms: Association with susceptibility to Henoch-Schönlein purpura and renal involvement | 114 | 7.2 ± 2.3  (3.5 – 16) | 73 – 41 |
| Cakir M, 2006 (119) | Henoch-Schönlein purpura in north-eastern Turkey | 116 | 8.9±3.7 | 73 – 43 |
| Chen O, 2013 (120) | Henoch Schönlein Purpura in children: Clinical analysis of 120 cases | 120 | 6.6 ±1.6 (1-12) | 78 - 42 |
| Nalbantoglu S, 2013 (121) | Association between RAS gene polymorphisms (ACE I/D, AGT M235T) and Henoch-Schönlein purpura in a Turkish population | 139 | 8.6 ± 3.1 | 77 – 62 |
| Zhao Y, 2015 (122) | Obesity increases the risk of renal involvement in children with Henoch-Schönlein purpura. | 141 | 7.8 ± 2.6 y  (2 – 14) | 76 – 65 |
| Mir S, 2007 (123) | Clinical outcome in children with Henoch-Schönlein nephritis | 141  82/141* | 8.9±3.29(2-17)  9.3±3.14(2-16) | 78 – 63  45 – 37 |
| Liu D, 2010 (124) | Renin-angiotensin system gene polymorphisms in children with Henoch-Schönlein purpura in West China | 142 | 9.03 (med) ± 2.85 | 81 – 61 |
| de Almeida J, 2007 (125) | Renal involvement in Henoch-Schönlein purpura: a multivariate analysis of initial prognostic factors | 142  70/142* | 6.1 ± 3.0  6.7 ± 2.9* | 66 – 76  35 – 35 |
| Trapani s, 2005 (126) | Henoch Schönlein purpura in childhood: Epidemiological and clinical analysis of 150 cases over a 5-year period and review of literature | 150 | 6.1 ± 2.7, 5.3 | 95 – 55 |
| Nussinovitch M, 1998 (127) | Cutaneous manifestations of Henoch-Schönlein purpura in young children | 155 | 6.8 ± 2.3 | 86 – 69 |
| Mollica F, 1992 (128) | Effectiveness of early prednisone treatment in preventing the development of nephropathy in anaphylactoid purpura | 156  12* | 6.3 ± 3.1  5.9 ± 2.1 | 67 – 89  5 – 7 |
| Aalberse J, 2007 (129) | Henoch-Schönlein purpura in children: an epidemiological study among Dutch paediatricians on incidence and diagnostic criteria | 179 | 6y (mean) 0 – 18 | 107 – 72 |
| Shin J, 2006 (130) | Predictive factors for nephritis, relapse, and significant proteinuria in childhood Henoch-Schönlein purpura | 206  128/206  78/206* | 7.2 ± 2.8  6.7 ± 2.6  8.2 ± 3.1* | 113 – 93 |
| Chen S, 2004 (131) | Gastrointestinal manifestations and complications of Henoch-Schönlein purpura | 208 | 6.4 (9m-15) | 116 – 92 |
| Bayrakci U, 2007 (132) | Effect of early corticosteroid therapy on development of Henoch-Schönlein nephritis | 216 | 8.45 ± 3.8 (3-19) | 121 – 95 |
| Dudley J, 2013 (133) | Randomized, double-blind, placebo-controlled trial to determine whether steroids reduce the incidence and severity of nephropathy in Henoch-Schönlein Purpura | 250 | 6 (med)  4 -8y (IQR) | 193 - 57 |
| Peru H, 2008 (134) | Henoch Schönlein purpura in childhood: Clinical analysis of 254 cases over a 3-year period | 254 | 8.65 ± 3.59 y | 147 – 107 |
| Prais D, 2007 (135) | Recurrent Henoch-Schönlein Purpura in Children | 260 | 5.7±2.6(1-14.8) | 148 - 112 |
| Chang W-L, 2005 (136) | Renal manifestations in Henoch-Schönlein purpura: A 10-year clinical study | 261  53/261* | 6.9 ± 3.5 y  8.6 ± 4.7 y | 137-124  26 – 27 |
| He X, 2012 (137) | C1GALT1 polymorphisms are associated with Henoch-Schönlein purpura nephritis | 269  61* | 7.16 ± 2.55  8.36 ± 2.75 | 173 – 96  44 – 17 |
| Calvo-Río V, 2014 (138) | Henoch-Schönlein Purpura in Northern Spain Clinical Spectrum of the Disease in 417 Patients from a Single Center | 315 | 7.5 y (med) | 170 - 145 |
| Lin Q, 2012 (139) | Henoch-Schönlein purpura with hipocomplementemia | 338 | 7.2 ± 2.4 y | 204 - 134 |
| Deng F, 2010 (140) | Henoch-Schönlein purpura in childhood: Treatment and prognosis. Analysis of 425 cases over a 5-year period | 425 | 9.0 ± 2.53  (2-14) | 270 - 155 |
| Liu A, 2012 (141) | Detection of antiphospholipid antibody in children with Henoch-Schönlein purpura and *central nervous system involvement | 426  46*(10%) | 7.85± 3.68  7.28 ± 2.35 | 237 -189  19 – 27 |
| Anil M, 2009 (142) | Henoch-Schönlein purpura in children from western Turkey: A retrospective analysis of 430 cases | 430 | 7.9 ± 2.9 y  (2 – 14y) | 225 - 205 |
| Gardner-Medwin J, 2002 (143) | Incidence of Henoch-Schönlein purpura, Kawasaki disease, and rare vasculitides in children of different ethnic origins | 463 | 6.4 | 1.2 - 1 |
| Weiss P, 2009 (144) | Variation in inpatient therapy and diagnostic evaluation of children with Henoch Schönlein purpura | 1988 | 6 med (0 – 17)  90% 2 – 15y | 1775 - 813 |
| Yang Y, 2015 (145)  ***In this article we are taking into account 10 references | Chinese herbal medicine for Henoch-Schönlein purpura in children without renal damage: A systematic review of randomized controlled trials  Trials were included when they met the following criteria: i) randomized controlled trials (RCTs) without limits on blinding or publishing language, ii) children with specified diagnosis criteria of HSP, no limits on the gender or nation  Trials with patients with renal damage and those included adult patients (aged over 18 years old) were excluded. | Chen 2012  Deng 2011  Dong 2003  Hou 2012  Huang 2013  Liu 2013  Yuan 2011  Yuan 2014  Zhao 2009 | 29, 8.3 ± 3.6  30, 7.22 ± 2.41  68, 8.4 (4-14)  30, 10.8 ± 4.8  29, 7.4 ± 1.5  45, 8.7 ± 4.1  32, 7.44 ±3.23  50, 10.5 ± 1.5  60, 7.5 ± 2.8 | 17 – 12  18 – 12  30 – 38  18 – 12  18 – 11  26 – 19  18 – 14  28 – 22  33 – 27 |

*Patients with nephritis were not included in the analysis. Pat #: Number of subjects included in the article. M – F: Number of males and females in the study.

References

1. Trapani S, Mariotti P, Resti M, Nappini L, De Martino M, Falcini F. Severe hemorrhagic bullous lesions in Henoch Schonlein purpura: Three pediatric cases and review of the literature. Rheumatol Int. 2010;30:1355–9.

2. Kawasaki K, Komura H, Nakahara Y, Shiraishi M, Higashida M, Ouchi K. Factor XIII in Henoch-Schönlein purpura with isolated gastrointestinal symptoms. Pediatr Int. 2006;48:413–5.

3. Gultekin A, Turkey S, Isik O. Intestinal Obstruction Due to Henoch-Schonlein Purpura. Indian Pediatr. 1989;26:72–4.

4. Belman AL, Leicher CR, Moshe SL, Mezey AP. Neurologic manifestations of Schoenlein-Henoch purpura: report of three cases and review of the literature. Pediatrics. 1985;75:687–92.

5. Somekh E, Fried D, Hanukoglu A. Muscle involvement in Schönlein-Henoch syndrome. Arch Dis Child. 1983;58:929–30.

6. Otieno LS, Lore W, Mureithi CJM. Schonlein-Henoch Syndrome with Nephropathy. East Afr Med J. 1982;59:352–6.

7. Zhang Y, Gu W, Mao J. Sibling cases of Henoch-Schönlein purpura in two families and review of literature. Pediatr Dermatol. 2008;25:393–5.

8. Chen YH, Lin TY, Chen CJ, Chen LK, Jan RH. Familial cases of Henoch-Schönlein purpura in Taiwanese aborigines. Pediatr Neonatol. Elsevier Taiwan LLC; 2012;53:320–4.

9. Casonato A, Pontara E, Bertomoro A, Ossi E, Vincenti M, Girolami A, et al. Abnormally Large von Willebrand Factor Multimers in Henoc h-Schon lein Pu rpu ra. Am J Hematol. 1996;1:7–11.

10. Sönmez K, Turkyilmaz Z, Demirogullari B, Karabulut R, Aral YZ, Konuş Ö, et al. Conservative treatment for small intestinal intussusception associated with Henoch-Schönlein’s purpura. Surg Today. 2002;32:1031–4.

11. Kato S, Shibuya H, Naganuma… H. Gastrointestinal endoscopy in Henoch-Schönlein purpura. Eur J Pediatr. 1992;151:482–4.

12. Coovadia HM. Henoch-Schönlein purpura in Black and Indian children in Natal. S Afr Med J. 1982;62:433–4.

13. Shin JI, Park JM, Shin YH, Lee JS, Jeong HJ. Role of mesangial fibrinogen deposition in the pathogenesis of crescentic Henoch-Schönlein nephritis in children. J Clin Pathol. 2005;58:1147–51.

14. Tomomasa T, Hsu JY, Ithoh K, Kuroume T. Endoscopic Findings in Pediatric Patients with Henoch-Schönlein Purpura and Gastrointestinal Symptoms. J Pediatr Gastroenterol Nutr. 1987;6:725–9.

15. Shin JI, Song KS, Kim H, Cho NH, Kim J, Kim HS, et al. The gene expression profile of matrix metalloproteinases and their inhibitors in children with Henoch-Schönlein purpura. Br J Dermatol. 2011;164:1348–55.

16. Motoyama O, Iitaka K. Henoch-Schönlein purpura with hypocomplementemia in children. Pediatr Int. 2005;47:39–42.

17. Dursun I, Düsünsel R, Poyrazoglu HM, Gunduz Z, PatIroglu T, Ülger H, et al. Circulating endothelial microparticles in children with Henoch-Schönlein purpura; Preliminary results. Rheumatol Int. 2011;31:1595–600.

18. Harada T, Kaneko T, Ito S, Hataya H, Nariai A, Mori M, et al. Superior mesenteric artery syndrome: Risk factor for duodenal involvement in Henoch-Schönlein purpura. Pediatr Int. 2011;53:630–3.

19. Kawasaki Y, Hosoya M, Suzuki H. Possible pathologenic role of interleukin-5 and eosino cationic protein in Henoch-Schönlein purpura nephritis. Pediatr Int. 2005;47:512–7.

20. Mahajan V, Singh S, Khullar M, Minz RW. Serum and urine nitric oxide levels in children with Henoch-Schonlein purpura during activity and remission: A study from north India. Rheumatol Int. 2009;29:1069–72.

21. Salhan M, Ahluwalia J, Singh S, Minz RW. Antiphospholipid antibodies in children with Henoch–Schonlein purpura: a prospective study from North India. Scand J Rheumatol. 2007;36:482–4.

22. Nishida M, Kawakatsu H, Ishiwari K, Tamai M, Sawada T, Nishimura M, et al. Serum hepatocyte growth factor levels in Henoch-Schönlein purpura. Pediatr Int. 1999;41:474–6.

23. Couture A, Veyrac C, Baud C, Galifer RB, Armelin I. Evaluation of abdominal pain in Henoch-Schönlein syndrome by high frequency ultrasound. Pediatr Radiol. 1992;22:12–7.

24. Tönshoff B, Momper R, Schweer H, Schärer K, Seyberth HW. Increased biosynthesis of vasoactive prostanoids in Schönlein-Henoch purpura. Pediatr Res. 1992;32:137–40.

25. Kagimoto S. Duodenal Findings on Ultrasound in children with Schonlein-Henoch Purpura and Gastrointestinal Symptoms. J Pediatr Gastroenterol Nutr. 1993;16:178–82.

26. Soylu A, Kizildaǧ S, Kavukçu S, Cingöz S, Türkmen M, Demir BK, et al. TLR-2 Arg753Gln, TLR-4 Asp299Gly, and TLR-4 Thr399Ile polymorphisms in Henoch Schonlein purpura with and without renal involvement. Rheumatol Int. 2010;30:667–70.

27. Motoyama O, Iitaka K. Familial cases of Henoch-Schönlein purpura in eight families. Pediatr Int. 2005;47:612–5.

28. Cazzato S, Bernardi F, Cinti C, Tassinari D, Canzi A, Bergamaschi R, et al. Pulmonary function abnormalities in children with Henoch-Schönlein purpura. Eur Respir J. 1999;13:597–601.

29. Gattorno M, Picco P, Barbano G, Stalla F, Sormani MP, Buoncompagni A, et al. Differences in Tumor Necrosis Factor-alpha Serum Concentrations Between Patients with Henoch-Schönlein Purpura and Pediatric Systemic Lupus Erythematosus: Pathogenic Implications. J Rheumatol. 1998;25:361–5.

30. Demircin G, Öner A, Ünver Y, Bülbül M, Erdogan O. Erythrocyte superoxide dismutase activity and plasma malondialdehyde levels in children with Henoch Schönlein purpura. Acta Paediatr. 1998;87:848–52.

31. De Mattia D, Penza R, Giordano P, GC DV, Aceto G, Altomare M, et al. von Willebrand factor and factor XIII in children with Henoch-Schönlein purpura. Pediatr Nephrol. 1995;9:603–5.

32. Farley TA, Gillespie S, Rasoulpour M, Tolentino N, Hadler JL, Hurwitz E. Epidemiology of a Cluster of Henoch-Schönlein Purpura. Am J Dis Child. 1989;143:798–803.

33. Brendel-Müller K, Hahn A, Schneppenheim R, Santer R. Laboratory signs of activated coagulation are common in Henoch-Schönlein purpura. Pediatr Nephrol. 2001;16:1084–8.

34. Henriksson P, Hedner U, Nilsson IM. Factor XIII (Fibrin Stabilising Factor) in Henoch-Schönlein Purpura. Acta Paediatr Scand. 1977;66:273–7.

35. Tsuji Y, Abe Y, Hisano M, Sakai T. Urinary leukotriene E4 in Henoch-Schonlein purpura. Clin Exp Allergy. 2004;34:1259–61.

36. Saulsbury FT, Kesler RW. Thrombocytosis in Henoch-Schönlein Purpura. Clin Pediatr. 1983;22:186–7.

37. Fuentes Y, Hernández AM, García-Roca P, Valverde S, Velásquez-Jones LF, Sosa G, et al. Urinary MCP-1/creatinine in Henoch-Schönlein purpura and its relationship with nephritis. Pediatr Nephrol. 2014;29:1047–52.

38. Mahajan N, Kapoor D, Bisht D, Singh S, Minz RW, Dhawan V. Levels of interleukin-18 and endothelin-1 in children with Henoch-Schönlein purpura: A study from Northern India. Pediatr Dermatol. 2013;30:695–9.

39. Pelliccia P, Natoli C, Petitti MT, Verrotti A, Chiarelli F, Iacobelli S. Elevated levels of circulating immunostimulatory 90K in Henoch-Schoenlein purpura. J Clin Immunol. 1999;19:143–7.

40. Moja P, Quesnel A, Resseguier V, Lambert C, Freycon F, Berthoux F, et al. Is there IgA from gut mucosal origin in the serum of children with Henoch-Schönlein purpura? Clin Immunol Immunopathol. 1998;86:290–7.

41. Söylemezoǧlu O, Sultan N, Gursel T, Buyan N, Hasanoglu E. Circulating adhesion molecules ICAM-1, E-selectin, and von Willebrand factor in Henoch-Schönlein purpura. Arch Dis Child. 1996;75:507–11.

42. Ozdemir H, Isik K, Buyan N, Hasanoglu E. Sonographic demonstration of intestinal involvement in Henoch-Schönlein syndrome. Eur J Radiol. 1995;20:32–4.

43. Huber AM, King J, McLaine P, Klassen T, Pothos M. A randomized, placebo-controlled trial of prednisone in early Henoch Schönlein Purpura [ISRCTN85109383]. BMC Med. 2004;2:7.

44. Cayci FS, Ekim M, Egin Y, Gökce H, Yalcinkaya F, Ozcakar B, et al. An Analysis of the Levels of the Soluble Form of the Endothelial Protein C Receptor in Children with Henoch-Schönlein Purpura. Pediatr Hematol Oncol. 2015;32:DOI: 10.3109/08880018.2013.860648.

45. Xie J, Liu Y, Wang L, Ruan G, Yuan H, Fang H, et al. Expansion of Circulating T Follicular Helper Cells in Children with Acute Henoch-Schönlein Purpura. J Immunol Res. 2015;2015(Article ID 742535):doi: /10.1155/2015/742535.

46. Aliyazicioglu Y, Ozkaya O, Yakut H, Islek I, Alvur M. Leptin levels in Henoch-Schönlein purpura. Clin Rheumatol. 2007;26:371–5.

47. Allen AC, Willis FR, Beattie TJ, Feehally J. Abnormal IgA glycosylation in Henoch-Schönlein purpura restricted to patients with clinical nephritis. Nephrol Dial Transpl. 1998;13:930–4.

48. Glasier CM, Siegel MJ, McAlister WH, Shackelford GD. Henoch-Schonlein Syndrome in Children: Gastrointestinal manifestations. Am J Roentgenol. 1981;136:1081–5.

49. Yilmaz A, Emre S, Agachan B, Bilge I, Yilmaz H, Ergen A, et al. Effect of paraoxonase 1 gene polymorphisms on clinical course of Henoch-Schönlein purpura. J Nephrol. 2009;22:726–32.

50. Del Vecchio GC, Penza R, Altomare M, Piacente L, Aceto G, Lassandro G, et al. Cytokine Pattern and Endothelium Damage Markers in Henoch-Schönlein Purpura. Immunopharmacol Immunotoxicol. 2008;30:623–9.

51. Fujieda M, Oishi N, Naruse K, Hashizume M, Nishiya K, Kurashige T, et al. Soluble thrombomodulin and antibodies to bovine glomerular endothelial cells in patients with Henoch-Schönlein purpura. Arch Dis Child. 1998;78:240–4.

52. Tahan F, Dursun I, Poyrazoglu H, Gurgoze M, Dusunsel R. The role of chemokines in Henoch Schonlein Purpura. Rheumatol Int. 2007;27:955–60.

53. Besbas N, Erbay A, Saatci Ü, Ozdemir S, Bakkaloglu A, Ozen S, et al. Thrombomodulin, tíssue plasminogen activator and plas ­ minogen activator inhibitor-l in Henoch-Schönlein purpura. Clin Exp Rheumatol. 1998;16:95–8.

54. Ece A, Kelekçi S, Kocamaz H, Hekimoǧlu A, Balik H, Yolbaş I, et al. Antioxidant enzyme activities, lipid peroxidation, and total antioxidant status in children with Henoch-Schönlein purpura. Clin Rheumatol. 2008;27:163–9.

55. Yang YH, Lai HJ, Kao CK, Lin YT, Chiang BL. The association between transforming growth factor-beta gene promoter C-509T polymorphism and Chinese children with Henoch-Schönlein purpura. Pediatr Nephrol. 2004;19:972–5.

56. Chaussain M, de Bolssieu D, Kalifa G, Epelbaum S, Niaudet P, Badoual J, et al. Impairment of lung diffusion capacity in Schönlein-Henoch purpura. J Pediatr. 1992;121:12–6.

57. Li Y, Li C, Wang G, Yang J, Zu Y. Investigation of the change in CD4+ T cell subset in children with Henoch-Schonlein purpura. Rheumatol Int. 2012;32:3785–92.

58. Wang YB, Shan NN, Chen O, Gao Y, Zou X, Wei DE, et al. Imbalance of interleukin-18 and interleukin-18 binding protein in children with Henoch-Schönlein purpura. J Inter Med Res. 2011;39:2201–8.

59. Fessatou S, Nicolaidou P, Gourgiotis D, Georgouli H, Douros K, Moustaki M, et al. Endothelin 1 levels in relation to clinical presentation and outcome of Henoch Schonlein purpura. BMC Pediatr. 2008;8:33.

60. Grover N, Sankhyan N, Bisht JP. A five year review of clinical profile in HSP. J Nepal Med Assoc. 2007;46:62–5.

61. Muslu A, Islek I, Gok F, Aliyazicioglu Y, Dagdemir A, Dundaroz R, et al. Endothelin levels in Henoch-Schonlein purpura. Pediatr Nephrol. 2002;17:920–5.

62. Kauffmann RH, Herrmann WA, Meÿer CJL, Daha MR, Van Es LA. Circulating IgA-Immune Complexes in Henoch-Schönlein Purpura. Am J Med. 1980;69:859–66.

63. Koskimies O, Mir S, Rapola J, Vilska J. Henoch-Schönlein Nephritis: Long-Term Prognosis of Unslected Patients. Arch Dis Child. 1981;56:482–4.

64. Qin W, Hongya W, Yongjing C, Fang X, Yue M, Xuekun Z, et al. Increased OX40 and soluble OX40 ligands in children with Henoch-Schonlein purpura: Association with renal involvement. Pediatr Allergy Immunol. 2011;22:54–9.

65. Zampetti A, Rigante D, Bersani G, Rendeli C, Feliciani C, Stabile A. Longitudinal study of microvascular involvement by nailfold capillaroscopy in children with Henoch-Schönlein purpura. Clin Rheumatol. 2009;28:1101–5.

66. Johnson EF, Lehman JS, Wetter DA, Lohse CM, Tollefson MM. Henoch-Schönlein purpura and systemic disease in children: Retrospective study of clinical findings, histopathology and direct immunofluorescence in 34 paediatric patients. Br J Dermatol. 2014;172:1358–63.

67. Ge W, Wang HL, Sun RP. Pentraxin 3 as a novel early biomarker for the prediction of Henoch-Schönlein purpura nephritis in children. Eur J Pediatr. 2014;173:213–8.

68. Eisenstein EM, Choi M. Analysis of a uteroglobin gene polymorphism in childhood Henoch-Schonlein purpura. Pediatr Nephrol. 2006;21:782–4.

69. Topaloglu R, Sungur A, Baskin E, Besbas N, Saatci U, Bakkaloglu A. Vascular Endothelial Growth Factor in Henoch-Schönlein Purpura. J Rheumatol. 2001;28:2269–73.

70. Chen T, Guo Z-P, Zhang Y-H, Gao Y, Liu H-J, Li J-Y. Elevated serum heme oxygenase-1 and insulin-like growth factor-1 levels in patients with Henoch-Schonlein purpura. Rheumatol Int. 2011;31:321–6.

71. Ozaltin F, Besbas N, Uckan D, Tuncer M, Topaloglu R, Ozen S, et al. The role of apoptosis in childhood Henoch-Schonlein purpura. Clin Rheumatol. 2003;22:265–7.

72. Saulsbury FT. Increased Serum IgD concentrations in children with Henoch-Schönlein purpura. Br J Rheumatol. 1998;37:570–2.

73. Blanco Quiros A, Blanco C, Alvarez J, Solis JAP, Gomez F, Conde S. Anti-immunoglobulin antibodies in children with Schönlein-Henoch syndrome. Absence of serum anti-lgA antibodies. Eur J Pediatr. 1994;153:103–6.

74. Inoue CN, Nagasaka T, Matsutani S, Ishidoya M, Homma R, Chiba Y. Efficacy of early dental and ENT therapy in preventing nephropathy in pediatric Henoch-Schönlein purpura. Clin Rheumatol. 2008;27:1489–96.

75. Al Rasheed SA, Adburrahman MB, Al Migeiren MM, Al Fawaz IM. Henoch-Schönlein Syndrome in Saudi Arabia. J Trop Pediatr. 1991;37:127–30.

76. Emre S, Sirin A, Ergen A, Bilge I, Sucu A, Yilmaz A, et al. Methylenetetrahydrofolate reductase C677T polymorphism in patients with Henoch-Schönlein purpura. Pediatr Int. 2011;53:358–62.

77. Nchimi A, Khamis J, Paquot I, Bury F, Magotteaux P. Significance of bowel wall abnormalities at ultrasound in Henoch-Schönlein purpura. J Pediatr Gastroenterol Nutr. 2008;46:48–53.

78. Yildiz B, Kural N, Aydin B, Colak O. Increased serum levels of insulin-like growth factor (IGF)-1 and IGF-binding protein-3 in Henoch-Schonlein purpura. Tohoku J Exp Med. 2008;214:333–40.

79. Kumar L, Singh S, Goraya JS, Uppal B, Kakkar S, Walker R, et al. Henoch-Schonlein purpura: The Chandigarh experience. Indian Pediatr. 1998;35:19–25.

80. Ilan Y, Naparstek Y. Schönlein- Henoch Syndrome in Adults and Children. Semin Arthritis Rheum. 1991;21:103–9.

81. Bagga A, Kabra SK, Srivastava RN, Bhuyan UN. Henoch-Schonlein Syndrome in Northern Indian Children. Indian Pediatr. 1991;28:1153–7.

82. Spasojević-Dimitrijeva B, Kostić M, Peco-Antić A, Kruščić D, Cvetković M, Miloševski-Lomić G, et al. Henoch-Schönlein purpura outcome in children: Ten-year clinical study. Srp Arh Celok Lek. 2011;139:174–8.

83. Mao YN, Liu W, Li YG, Jia GC, Zhang Z, Guan YJ, et al. Urinary angiotensinogen levels in relation to renal involvement of Henoch-Schonlein purpura in children. Nephrology. 2012;17:53–7.

84. Dagan E, Brik R, Broza Y, Gershoni-Baruch R. Henoch-Schonlein purpura: Polymorphisms in thrombophilia genes. Pediatr Nephrol. 2006;21:1117–21.

85. Gershoni-Baruch R, Broza Y, Brik R. Prevalence and significance of mutations in the familial mediterranean fever gene in Henoch-Schönlein Purpura. J Pediatr. 2003;143:658–61.

86. Albaramki J. Henoch-Schonlein Purpura in Childhood a Fifteen-Year Experience At a Tertiary Hospital. J Med Liban. 2016;64:13–7.

87. Alfredo CS, Nunes N a, Len C a, Barbosa CMP, Terreri MTR a, Hilário MOE. Henoch-Schönlein purpura: recurrence and chronicity. J Pediatr (Rio J). 2007;83:177–80.

88. Salah S, Rizk S, Lotfy HM, El Houchi S, Marzouk H, Farag Y. MEFV gene mutations in Egyptian children with Henoch-Schonlein purpura. Pediatr Rheumatol Online J. 2014;12:41.

89. Nickavar A, Mehrazma M, Lahouti A. Clinicopathologic correlations in Henoch-Schonlein nephritis. Iran J Kidney Dis. 2012;6:437–40.

90. Smith G, Davidson J, Hughes D. Complement activation in Henoch-Schönlein Purpura. Pediatr Nephrol. 1997;11:477–80.

91. Hung SP, Yang YH, Lin YT, Wang LC, Lee JH, Chiang BL. Clinical Manifestations and Outcomes of Henoch-Schönlein Purpura: Comparison between Adults and Children. Pediatr Neonatol. Taiwan Pediatric Association; 2009;50:162–8.

92. Kanik A, Baran M, Ince FD, Cebeci O, Bozkurt M, Cavusoglu D, et al. Faecal calprotectin levels in children with Henoch–Schönlein purpura: is this a new marker for gastrointestinal involvement? Eur J Gastroenterol Hepatol. 2015;27:254–8.

93. Pisacane A, Buffolano W, Grillo G, Gaudiosi C. Infant Feeding and Henoch-Schönlein Purpura. Acta Paeditr. 1992;81:630-.

94. Qin YH, Zhou TB, Lei FY, Huang WF, Zhao YJ, Lin FQ, et al. Cut-off values for serum matrix metalloproteinase-9: Is there a threshold to predict renal involvement for Henoch-Schonlein purpura in children? Nephrology. 2011;16:93–9.

95. Hamdan JM, Barqawi MA. Henoch-Schonlein purpura in children. Influence of age on the incidence of nephritis in children. Saudi Med J. 2008;29:549–52.

96. Martínez López MM, Rodríguez Arranz C, Peña Carrión A, Merino Muñoz R, García-Consuegra Molina J. Púrpura de Schönlein-Henoch. Estudio de factores asociados con el desarrollo y evolución de la enfermedad. An Pediatr. 2007;66:453–8.

97. Wang X, Zhu Y, Gao L, Wei S, Zhen Y, Ma Q. Henoch-Schönlein purpura with joint involvement: Analysis of 71 cases. Pediatr Rheumatol. Pediatric Rheumatology; 2016;14:20.

98. García-Porrúa C, Calviño MC, Llorca J, Couselo JM, González-Gay MA. Henoch-Schönlein purpura in children and adults: Clinical differences in a defined population. Semin Arthritis Rheum. 2002;32:149–56.

99. Lardhi AA. Henoch-Schonlein purpura in children from the eastern province of Saudi Arabia. Saudi Med J. 2012;33:973–8.

100. Ding W, Sun S, Zhen J, Yu Y, Wang Y. Urinary excretion and renal production of hepatocyte growth factor in children with Henoch-Schönlein purpura. J Inter Med Res. 2010;38:1933–41.

101. Calviño MC, Llorca J, García-Porrúa C, Fernández-Iglesias JL, Rodríguez-Ledo P, González-Gay MA. Henoch-Schönlein purpura in children from northwestern Spain: a 20-year epidemiologic and clinical study. Medicine (Baltimore). 2001;80:279–90.

102. Elmas AT, Tabel Y. Platelet Counts in Children With Henoch-Schonlein Purpura-Relationship to Renal Involvement. J Clin Lab Anal. 2016;30:71–4.

103. Wu SH, Liao PY, Chen XQ, Yin PL, Dong L. Add-on therapy with montelukast in the treatment of Henoch-Schönlein purpura. Pediatr Int. 2014;56:315–22.

104. Ronkainen J, Koskimies O, Ala-Houhala M, Antikainen M, Merenmies J, Rajantie J, et al. Early prednisone therapy in Henoch-Schonlein purpura: a randomized, double-blind, placebo-controlled trial. J Pediatr. 2006;149:241–7.

105. Lin SJ, Huang JL. Henoch-Schönlein purpura in Chinese children and adults. Asian Pac J Allergy Immunol. 1998;16:21–5.

106. Yang YH, Chang CJ, Chuang YH, Hsu HY, Yu HH, Lee JH, et al. Identification and characterization of IgA antibodies against B2-glycoprotein I in childhood Henoch-Schönlein purpura. Br J Dermatol. 2012;167:874–81.

107. Teng X, Wang Y, Lin N, Sun M, Wu J. Evaluation of serum procalcitonin and C-reactive protein levels as biomarkers of Henoch-Schönlein purpura in pediatric patients. Clin Rheumatol. 2016;35:667–71.

108. Cohen N, Mimouni FB, Friedel N, Amarilyo G. Predictors of hospital length of stay in pediatric Henoch-Schönlein purpura. Rheumatol Int. Springer Berlin Heidelberg; 2015;35:1561–4.

109. Rigante D, Candelli M, Federico G, Bartolozzi F, Porri MG, Stabile A. Predictive factors of renal involvement or relapsing disease in children with Henoch-Schönlein purpura. Rheumatol Int. 2005;25:45–8.

110. Kawasaki Y, Suzuki J, Sakai N, Nemoto K, Nozawa R, Suzuki S, et al. Clinical and pathological features of children with Henoch-Schoenlein purpura nephritis: risk factors associated with poor prognosis. Clin Nephrol. 2003;60:153–60.

111. Xu H, Li W, Fu H, Jiang G. Interferon-Gamma Gene Polymorphism + 874 ( A / T ) in Chinese Children with Henoch-Schönlein Purpura. Iran J Allergy Asthma Immunol. 2014;13:184–9.

112. Soylemezoglu O, Peru H, Gonen S, Cetinyurek A, Ozkaya O, Bakkaloǧlu S, et al. CTLA-4 +49 A/G genotype and HLA-DRB1 polymorphisms in Turkish patients with Henoch-Schönlein purpura. Pediatr Nephrol. 2008;23:1239–44.

113. Saulsbury FT. Henoch-Schönlein purpura in children. Report of 100 patients and review of the literature. Medicine (Baltimore). 1999;78:395–409.

114. Watson L, Richardson ARW, Holt RCL, Jones CA, Beresford MW. Henoch Schonlein purpura - A 5-year review and proposed pathway. PLoS One. 2012;7:e2951; doi:10.1371/journal.pone.0029512.

115. Bayram C, Demircin G, Erdoǧan Ö, Bülbül M, Çaltik A, Akyüz SG. Prevalence of MEFV gene mutations and their clinical correlations in Turkish children with Henoch-Schönlein purpura. Acta Paediatr. 2011;100:745–9.

116. Shin J Il, Lee SJ, Lee JS, Kim KH. Intravenous dexamethasone followed by oral prednisolone versus oral prednisolone in the treatment of childhood Henoch–Schönlein purpura. Rheumatol Int. 2011;31:1429–32.

117. Kang Y, Park J, Ha Y, Kang M, Park H-J, Lee S-W, et al. Differences in clinical manifestations and outcomes between adult and child patients with Henoch-Schönlein purpura. J Korean Med Sci. 2014;29:198–203.

118. Özkaya O, Söylemezoǧlu O, Gönen S, Misirlioǧolu M, Tuncer S, Kalman S, et al. Renin-angiotensin system gene polymorphisms: Association with susceptibility to Henoch-Schonlein purpura and renal involvement. Clin Rheumatol. 2006;25:861–5.

119. Cakir M, Orhan F, Mungan I, Sonmez FM, Aslan Y, Kalyoncu M, et al. Henoch-Schönlein purpura in north-eastern Turkey. Ann Trop Paediatr. 2006;26:59–65.

120. Chen O, Zhu XB, Ren P, Wang YB, Sun R, Wei DE. Henoch Schonlein Purpura in children: Clinical analysis of 120 cases. Afr Heal Sci. 2013;13:94–9.

121. Nalbantoglu S, Tabel Y, Mir S, Serdaroǧlu E, Berdeli A. Association between RAS gene polymorphisms (ACE I/D, AGT M235T) and Henoch-Schönlein purpura in a Turkish population. Dis Markers. 2013;34:23–32.

122. Zhao Y-L, Liu Z-J, Bai X-M, Wang Y-C, Li G-H, Yan X-Y. Obesity increases the risk of renal involvement in children with Henoch-Schönlein purpura. Eur J Pediatr. 2015;174:1357–63.

123. Mir S, Yavascan O, Mutlubas F, Yeniay B, Sonmez F. Clinical outcome in children with Henoch-Schönlein nephritis. Pediatr Nephrol. 2007;22:64–70.

124. Liu D, Lu F, Zhai S, Wei L, Ma S, Chen X, et al. Renin-angiotensin system gene polymorphisms in children with Henoch-Schönlein purpura in West China. J Renin Angiotensin Aldosterone Syst. 2010;11:248–55.

125. de Almeida JLJ, Campos LM a, Paim LB, Leone C, Koch VHK, Silva CA a. Renal involvement in Henoch-Schönlein purpura: a multivariate analysis of initial prognostic factors. J Pediatr (Rio J). 2007;83:259–66.

126. Trapani S, Micheli A, Grisolia F, Resti M, Chiappini E, Falcini F, et al. Henoch Schonlein purpura in childhood: Epidemiological and clinical analysis of 150 cases over a 5-year period and review of literature. Semin Arthritis Rheum. 2005;35:143–53.

127. Nussinovitch M, Prais D, Finkelstein Y, Varsano I. Cutaneous manifestations of Henoch-Schönlein purpura in young children. Pediatr Dermatol. 1998;15:426–8.

128. Mollica F, LiVolti S, Garozzo R, Russo G. Effectiveness of early prednisone treatment in preventing the development of nephropathy in anaphylactoid purpura. Eur J Pediatr. 1992;151:140–4.

129. Aalberse J, Dolman K, Ramnath G, Pereira RR, Davin J-C. Henoch-Schönlein purpura in children: an epidemiological study among Dutch paediatricians on incidence and diagnostic criteria. Ann Rheum Dis. 2007;66:1648–50.

130. Shin JI, Park JM, Shin YH, Hwang DH, Kim JH, Lee JS. Predictive factors for nephritis, relapse, and significant proteinuria in childhood Henoch-Schönlein purpura. Scand J Rheumatol. 2006;35:56–60.

131. Chen S-Y, Kong M-S. Gastrointestinal manifestations and complications of Henoch-Schönlein purpura. Chang Gung Med. 2004;27:175–81.

132. Bayrakci US, Topaloglu R, Soylemezoglu O, Kalyoncu M, Ozen S, Besbas N, et al. Effect of early corticosteroid therapy on development of Henoch-Schonlein nephritis. J Nephrol. 2007;20:406–9.

133. Dudley J, Smith G, Llewelyn-Edwards A, Bayliss K, Pike K, Tizard J. Randomised, double-blind, placebo-controlled trial to determine whether steroids reduce the incidence and severity of nephropathy in Henoch-Schönlein Purpura (HSP). Arch Dis Child. 2013;98:756–63.

134. Peru H, Soylemezoglu O, Bakkaloglu SA, Elmas S, Bozkaya D, Elmaci AM, et al. Henoch Schonlein purpura in childhood: Clinical analysis of 254 cases over a 3-year period. Clin Rheumatol. 2008;27:1087–92.

135. Prais D, Amir J, Nussinovitch M. Recurrent Henoch-Schönlein Purpura in Children. J Clin Rheumatol. 2007;13:25–8.

136. Chang WL, Yang YH, Wang LC, Lin YT, Chiang BL. Renal manifestations in Henoch-Schönlein purpura: A 10-year clinical study. Pediatr Nephrol. 2005;20:1269–72.

137. He X, Zhao P, Kang S, Ding Y, Luan J, Liu Z, et al. C1GALT1 polymorphisms are associated with Henoch-Schönlein purpura nephritis. Pediatr Nephrol. 2012;27:1505–9.

138. Calvo-Río V, Loricera J, Mata C, Martín L, Ortiz-Sanjuán F, Alvarez L, et al. Henoch-Schönlein Purpura in Northern Spain Clinical Spectrum of the Disease in 417 Patients from a Single Center. Medicine (Baltimore). 2014;93:106–13.

139. Lin Q, Min Y, Li Y, Zhu Y, Song X, Xu Q, et al. Henoch-Schönlein purpura with hypocomplementemia. Pediatr Nephrol. 2012;27:801–6.

140. Deng F, Lu L, Zhang Q, Hu B, Wang SJ, Huang N. Henoch-Schönlein purpura in childhood: Treatment and prognosis. Analysis of 425 cases over a 5-year period. Clin Rheumatol. 2010;29:369–74.

141. Liu A, Zhang H. Detection of antiphospholipid antibody in children with Henoch-Schönlein purpura and central nervous system involvement. Pediatr Neurol. Elsevier Inc.; 2012;47:167–70.

142. Anil M, Aksu N, Kara OD, Bal A, Anil AB, Yavaşcan Ö, et al. Henoch-Schönlein purpura in children from western Turkey: A retrospective analysis of 430 cases. Turk J Pediatr. 2009;51:429–36.

143. Gardner-Medwin JMM, Dolezalova P, Cummins C, Southwood TR. Incidence of Henoch-Schönlein purpura, Kawasaki disease, and rare vasculitides in children of different ethnic origins. Lancet. 2002;360:1197–202.

144. Weiss PF, Klink AJ, Hexem K, Burnham JM, Leonard MB, Keren R, et al. Variation in inpatient therapy and diagnostic evaluation of children with Henoch Schönlein purpura. J Pediatr. Elsevier Inc.; 2009;155:812–8.

145. Yang Y, Wang C, Li X, Chai Q, Fei Y, Xia R, et al. Chinese herbal medicine for Henoch-Schönlein purpura in children without renal damage: A systematic review of randomized controlled trials. Complemen Ther Med. Elsevier Ltd; 2015;23:741–50.
